# The association between executive functioning and parental stress and psychological distress is mediated by parental reflective functioning in mothers with substance use disorder

**DOI:** 10.1002/smi.2868

**Published:** 2019-08-02

**Authors:** Ulrika Håkansson, Reidulf G. Watten, Kerstin Söderström, Merete Glenne Øie

**Affiliations:** ^1^ Department of Psychology Innland Norway University of Applied Sciences Lillehammer Norway; ^2^ Division of Mental Health Care Innlandet Hospital Trust Lillehammer Norway; ^3^ Department of Psychology University of Oslo Oslo Norway; ^4^ Research Division Innlandet Hospital Trust Lillehammer Norway

**Keywords:** executive functioning, maternal, parental reflective functioning, parental stress, psychological distress, substance use disorder

## Abstract

Mothers with a substance use disorder (SUD) have been found to exhibit heightened experience of stress and deficits in executive functioning (EF) and in parental reflective functioning (PRF). Although experiences of stress, EF and PRF are important for caregiving capacities; no studies have explored associations between the phenomena in mothers with SUD. This study aimed to examine the association between EF (working memory, inhibition, and cognitive flexibility) and different forms of stress (parental stress, general life stress, and psychological distress) in 43 mothers with SUD with infants. We further aimed to investigate whether PRF had a mediating function between EF and the experience of stress. The mothers completed self‐report questionnaires regarding experiences of different types of stress, and we also used neuropsychological tests to assess EF and a semistructured interview to assess PRF. Results identified problems in EF were associated with higher parental stress and psychological distress but not with general life stress. Cognitive flexibility contributed uniquely to variance in parental stress, whereas working memory was a unique contributor to variance in psychological distress. PRF had a mediating function between EF and parental stress and between EF and psychological distress. Findings highlight the importance of considering individual differences in PRF when targeting EF in interventions trying to reduce the experience of parental stress and psychological distress in mothers with SUD.

## 1 INTRODUCTION

Studies on mothers with substance use disorder (SUD) have demonstrated that as a group, these mothers exhibit high levels of stress (Kelley, 1998; Nair, Schuler, Black, Kettinger, & Harrington, 2003; Zvolensky & Hogan, 2013) and difficulties in stress‐regulation capacities (Tronick et al., 2005), compared with normal populations. The experience of stress is related to processes involved in perception, appraisal, and response to challenging or threatening stimuli (Sinha, 2008; Sinha et al., 2005). The perception and appraisal of stress depend on both internal and external conditions, emotional states, personality factors, and individual resources (Sinha, 2008). Mothers with low emotion regulation capacity with young children have been found to exhibit reduced distress tolerance (Deater‐Deckard, Li, & Bell, 2016), and in particular, mothers with SUD have a heightened risk for emotion dysregulation (Suchman, DeCoste, Ordway, & Mayes, 2012). Indeed, reduced distress tolerance is thought to be a central component of SUD (Li & Sinha, 2008; Tronick et al., 2005). Mothers with SUD may therefore be more vulnerable to stress exposure compared with mothers without SUD. Stress exposure may result from general life stress (e.g., divorce, loss of job, interpersonal conflict, or socio‐economic resources), psychological distress (e.g., stress related to mental health issues/SUD), or parental stress. Parental stress refers to the experience of distress or discomfort arising from the demands associated with parenting (Deater‐Deckard, 1998). Parental stress constitutes of stress related to the parents' appraisal of the child and stress related to experiences concerning the parental role (Abidin, 1995). Elevated levels of parenting stress in mothers with SUD may place their children at an increased risk due to dysfunctional parent–child relationship (Hans, Bernstein, & Henson, 1999; Nair et al., 2003). Mothers with higher ratings of psychological distress are more likely to perceive their infant's behaviour as stressful (Sheinkopf et al., 2005).

Research on individual differences in stress regulation has traditionally focused on phenotypic personality factors, based on childhood and environmental aspects (e.g., abuse and neglect in childhood, socio‐economic factors, and substance abuse in adulthood) and genotype (Williams, Suchy, & Rau, 2009). However, there has been a recent focus on elucidating the neurocognitive underpinnings of these individual differences. For instance, each of the processes involved in stress, which include exposure, reactivity, recovery, and restoration, is moderated by a set of cognitive processes known collectively as executive functioning (EF). EF is generally applied as an umbrella construct referring to a set of basic neurocognitive processes that facilitate conscious control of thoughts, actions, and emotions that together result in complex, goal‐directed behaviours, such as the ability to maintain and shift focus, monitor outcomes, and alter behaviours (Diamond, 2013; Zelazo, 2015). EF includes three main processes: working memory, inhibition, and cognitive flexibility (Diamond, 2013). Working memory is the ability to keep information in mind, update, and integrate current contents with new information. Inhibition is the ability to inhibit proponent responses in order to selectively attend to relevant information and engage in goal‐directed rather than habitual and/or impulsive, actions. Cognitive flexibility is the ability to shift between cognitive rules of modes (Friedman & Miyake, 2017). These basic facets of EF are thought to underlie successful emotion regulation (Hofmann, Schmeichel, & Baddeley, 2012). Mothers with SUD have a heightened risk to exhibit difficulties in emotion regulation capacities (Suchman et al., 2012) and experiencing emotion dysregulation in response to parental stress (Skowron, Kozlowski, & Pincus, 2010; Suchman et al., 2012). Research has found that poor emotional regulation capacities are related to reduction in distress tolerance (Deater‐Deckard et al., 2016), and a recent study found associations between EF and emotion regulation in women with SUD (Marceau, Kelly, & Solowij, 2018). Furthermore, EF is sensitive to sociodemographic factors; for instance, higher age and lower education levels are associated with lower EF (Campanholo et al., 2017). Therefore, individual differences in EF may influence the experience of stress and the capacity to manage stress and emotional dysregulation (Williams et al., 2009).

In a recent study, individual differences in EF are suggested to be dynamic and dependent on individual capacities in allocating limited cognitive resources when facing stress (Kluwe‐Schiavon, Viola, Sanvicente‐Vieira, Malloy‐Diniz, & Grassi‐Oliveira, 2016). A shift from controlled EF to more automatic processes during emotional dysregulation and the ability to affect regulation are dependent on the individual's capacity for adaptive use of existing EF resources (Gagnon & Wagner, 2016; Kluwe‐Schiavon et al., 2016). The unique individual EF profile could therefore be associated with stress‐regulation capacities.

Disruptions in a number of EF components are commonly found in individuals with SUD (Kalivas & Volkow, 2005). In addition, substance dependency is associated with neural abnormalities in the frontal lobes, which are brain areas linked to alterations in EF (Moreno‐López et al., 2012). Impairments in working memory (Bechara, Martin, & Becker, 2004), inhibition (Dolan, Bechara, & Nathan, 2008), and cognitive flexibility (Cunha, Nicastri, de Andrade, & Bolla, 2010) have been found both during substance use and during substance abstinence in individuals with SUD (Verdejo‐García, Bechara, Recknor, & Perez‐Garcia, 2006).

An effective EF system is thought to regulate parenting behaviour and to support the ability of perception, responsiveness, and flexibility in relation to parental demands (Kienhuis, Rogers, Giallo, Matthews, & Treyvaud, 2010). Furthermore, good enough EF is suggested to be a prerequisite for sensitive caregiving (Gonzalez, Jenkins, Steiner, & Fleming, 2012). Recently, EF and particularly cognitive flexibility and working memory have been associated with parental reflective functioning (PRF; Håkansson, Söderström, Watten, Skårderud, & Øie, 2017; Rutherford et al., 2017; Yatziv, Kessler, & Atzaba‐Poria, 2018). Reflective functioning (RF) is the operationalization of mentalizating, which is the ability to understand oneself and others in terms of feelings, wishes, and thoughts, in addition to having a capacity to interpret mental states as underlying behavioural expressions (Fonagy & Target, 1997). PRF is specifically related to mentalizing regarding ones' child, oneself as a parent, and the parent–child relationship (Slade, 2005). Prefrontal brain areas that are important for EF are areas also found to be involved with PRF (Abu‐Akel & Shamay‐Tsoory, 2011; Oldrati, Patricelli, Colombo, & Antonietti, 2016), which suggests a possible association between EF and PRF. Furthermore, mothers with SUD who have negative to low PRF have been found to exhibit weaker EF compared with mothers with adequate PRF (Håkansson et al., 2017). Low mentalizing capacities have been associated with elevated stress levels (Luyten, Fonagy, & Lowyck, 2012), whereas adequate PRF is associated with increased tolerance of child distress (Rutherford, Goldberg, Luyten, Bridgett, & Mayes, 2013). In addition, mothers with low PRF displayed decreased tolerance of distress on behavioural and self‐report measures and assessments for peripheral physiology (Rutherford, Booth, Luyten, Bridgett, & Mayes, 2015). Furthermore, low PRF heightens stress sensitivity in mothers with mental health problems (Krink, Muehlhan, Luyten, Romer, & Ramsauer, 2018).

It has been recommended that research on parenting, EF, and stress‐regulation in high‐risk groups of parents should be prioritized (Crandall, Deater‐Deckard, & Riley, 2015). Indeed, to our knowledge, there are no studies exploring the association between different types of stress, EF, and PRF in mothers with SUD with infants. Considering the potential influence these factors have on caregiving capacities, it is important to advance knowledge in this area in order to contribute to the development of effective clinical interventions to improve parenting capacities.

### 1.1 The current study

The purpose of the current study was to explore the complex association between EF (working memory, inhibition, and cognitive flexibility), PRF, and experience of stress (parental stress, general life stress, and psychological distress), among mothers with SUD with infants.

The study consists of two parts. In the first part, we used a correlational design to investigate the associations between EF (working memory, inhibition, and cognitive flexibility) and the experience of stress (parental stress, general life stress, and psychological distress). We aimed to investigate how EF components were associated with different experiences of stress. In addition, we wanted to investigate if particular EF components contributed to the variance in experienced stress more than others. We hypothesized that (a) there would be negative associations between EF components (working memory, inhibition, and cognitive flexibility) and experience of stress (parental stress, general life stress, and psychological distress) and (b) we expected low cognitive flexibility, inhibition, and working memory capacities to contribute to more experience of stress (parental stress, general life stress, and psychological distress).

In the second part of the study, we were interested in investigating PRF as a mediator between EF (working memory, inhibition, and cognitive flexibility) and the experience of stress. Therefore, we aimed to investigate the associations between EF and PRF and between PRF and the experience of stress. Furthermore, as research has indicated an association between EF and mentalizing and specifically that adequate PRF is associated with increased tolerance of child distress, we aimed to explore whether PRF had a mediating effect between EF (working memory, inhibition, and cognitive flexibility) and the experience of stress in mothers with SUD. We hypothesized that (a) EF would be positively associated with PRF and PRF would be negatively associated with stress and (b) PRF would have a mediating function between EF and the experience of stress.

## 2 METHOD

### 2.1 Participants

The sample for this study consisted of 43 mother–infant dyads. Inclusion criteria required that mothers (*M* = 31.0 years; *SD* = 6.4) had a SUD diagnosis and a child under the age of 18 months (*M* = 8.6 months; *SD* = 3.8). Mothers with or without a comorbid mental illness in addition to SUD were recruited during pregnancy or early during the postpartum period. Referrals were received from municipality nurses, clinicians in outpatient services, and clinicians in institutions specialized in caring for pregnant women with SUD. All the mothers were abstinent during the assessments. Eleven mothers (25.6%) received medical‐assisted treatment, using either Methadone or Buprenorphine during pregnancy. Exclusion criteria were estimated full IQ below 70 in the mother measured by the Wechsler Abbreviated Scale of Intelligence (Wechsler, 2014; Weschler, 1999), multiple birth (i.e., giving birth to twins or triplets), premature birth (<32 weeks and <1,500 g), or severely ill or multihandicapped child. Ten children (23.3%) were born with neonatal abstinence syndrome. Neonatal abstinence syndrome was not an exclusion criterion.

Average education of the mothers was 11.5 years (range 7 to 18 years). Two participants (4.7%) had not completed primary school, and 22 (51.2%) had started but not completed high school. Six participants (14.0%) had graduate or professional degree beyond high school. During the assessments, all the mothers were on paid maternity leave or paid sick leave due to SUD. Twenty‐two mothers (51. 2%) did not have a partner, and 13 (30.2%) had a cohabitant. One participant (2.3%) was married, and seven (16.3%) had a partner who was not a cohabitant. Although 16 of the mothers (37.2%) had older children, only one (2.3%) had custody of the older sibling of the participating child; therefore, we did not control for number of children in the household.

### 2.2 Measures

#### 2.2.1 Sociodemographic variables and use of psychoactive substances

Substance use and sociodemographic variables were registered with the European Addiction Severity Index (Europ‐ASI) Fifth Edition (Kokkevi & Hartgers, 1995; McLellan et al., 1992), Norwegian version (Lauritzen, 2010). Europ‐ASI is a semistructured clinical interview and consists of questions related to employment and support status, family and social relationship, legal and illegal substance use, and somatic and psychological issues. As all the mothers were abstinent during inclusion and assessment, we did not assign an ASI severity score. Reliability and validity for the Europ‐ASI have previously been reported to be satisfactory (Kessler et al., 2012; Kokkevi & Hartgers, 1995; McLellan et al., 1992). In the current study, the Cronbach *α* coefficient was .79, which was considered satisfying.

#### 2.2.2 Stress

We administered two self‐report questionnaires to assess parental stress, general life stress, and psychological distress.

##### Parental stress and general life stress

We used the Parenting Stress Index Third Edition (PSI, long form; Abidin, 1995) to assess for parental stress and general life stress. The PSI is a 120‐item inventory widely used self‐report assessment of three major sources of stress. The instrument measures the parent's subjective experience of (a) stress related to child characteristics and the parent's appraisal of them, (b) stress related to own appraisal of parental characteristics and family context variables that can compromise parenting, and (c) potential stressful circumstances outside the dyadic relationship, usually experienced as stressful. A total stress score may be derived from the sum of (a) child characteristics scale and (b) parental characteristics scale, referred to as *parental stress*. Stressful circumstances outside the dyad are referred to as *general life stress*, which is separately indexed from 19 items in the questionnaire. The majority of items are rated on a 5‐point Likert scale from 1 = *Strongly Disagree* to 5 = *Strongly Agree*. A few items are rated *Yes*/*No* depending on if they are present or absent. The manual provides percentile cut‐offs indicating adequate stress level <80th or a clinically high stress level ≥80th. The PSI has previously showed good test–retest reliability and good internal consistency (Abidin, 1995). In the current study, the Cronbach *α* coefficient was .85, indicating a good internal consistency for the scale within this sample of mothers.

##### Psychological distress

Hopkins Symptom Checklist (HSCL‐10) was used to measure psychological distress. HSCL‐10 is a self‐administered questionnaire that assesses subjective experiences of anxiety and depression symptoms. HSCL‐10 is a short version of the HSCL‐90 (Derogatis, Lipman, Rickels, Uhlenhuth, & Covi, 1974). The instrument consists of 10 items and responses ranges from 1 = *Not at all* to 4 = *Very much*. The average item score is calculated by dividing the total score of the number of items answered. A score of ≥1.85 is considered to indicate clinically high psychological distress (Strand, Dalgard, Tambs, & Rognerud, 2003). HSCL‐10 has previously been shown to have satisfactory validity and reliability (Haavet, Sirpal, Haugen, & Christensen, 2010; Strand et al., 2003). The HSCL‐10 had a Cronbach *α* coefficient of .83 in this sample of mothers, which indicates a strong internal consistency.

#### 2.2.3 Executive functioning

Neuropsychological measures of maternal EF included assessment of several executive subfunctions.

##### Working memory

The Letter‐Number Sequencing subtest from the Wechsler Adult Intelligence Scale Fourth Edition (Wechsler, 2014) was used to assess working memory. The participants were presented with increasingly longer series of mixed letters and numbers at 1‐s intervals and were asked to repeat the series back to the administrator with the numbers presented first, from lowest to highest, followed by the letters in alphabetical order. Higher raw *t* scores and longer spans are consistent with a high capacity of auditory working memory. The Wechsler Adult Intelligence Scale has satisfactory validity and reliability (Canivez & Watkins, 2010).

##### Cognitive inhibition

We used the Colour‐Word Interference Test, Condition 3 from the Delis–Kaplan Executive Function System (D‐KEFS; Delis, Kaplan, & Kramer, 2001) to assess cognitive inhibition. Participants were required to inhibit reading a colour‐word and instead say the name of the colour in which the word was printed as fast as possible. Higher frequency of errors, in addition to longer time to complete the task, indicated more difficulties with inhibition and provided a lower *t* score.

##### Cognitive flexibility

We administered the inhibition‐switching task in Colour‐Word Interference Test, Condition 4 from the D‐KEFS (Delis et al., 2001) to assess cognitive flexibility. Participants were asked to switch between reading the colour‐word and naming the colour in which the colour is printed. The time used and the number of errors committed during the task were measured. Longer time to complete the task in addition to having more errors indicates difficulties with cognitive flexibility and provided a lower *t* score. The D‐KEFS has good reliability and validity (Delis, Kramer, Kaplan, & Holdnack, 2004). In this sample of mothers, the D‐KEFS scale had a Cronbach *α* of .78, indicating a satisfying internal consistency.

#### 2.2.4 Parental reflective function

We used the Parent Development Interview‐Revised (PDI‐R2) to assess PRF (Fonagy, Target, Steele, & Steele, 1998; Slade et al., 2003), Norwegian translation. PDI‐R2 is a 20‐question semistructured interview developed to elicit narratives of parental representations of themselves, their child, and the relationship between them. The interview addresses various themes concerning the child's and the parent's feelings, thoughts, and intentions and how these might affect mental processes and behaviour and influence the person reflected upon (both in the parent herself, in the child, and in the mother's own parents). We recorded, transcribed, and coded the interview in accordance with the guidelines for RF assessment (Fonagy et al., 1998). An independent reliable coder who was not familiar with the respondents rated the interviews. A second independent coder rated 25% of the interviews for reliability purposes. There was a strong intraclass correlation between the coders (*r* = .96). In line with the guidelines, the interviews were scored for PRF on an 11‐point scale from −1 to 9, with higher scores reflecting a higher PRF (Slade, Bernbach, Grienenberger, Levy, & Locker, 2005). A score of −1 indicates negative PRF and includes a violation of coherence (bizarre) or openly hostile responses. A score of 9 indicates exceptionally high PRF, with responses that are rich and full of reflections. Validity for the PDI‐RF is satisfactory in nonclinical populations and in populations of parents with SUD (Levy & Truman, 2002; Slade, 2005; Slade, Belsky, Aber, & Phelps, 2005).

### 2.3 Procedures

Participants were assessed either at their own home or in the treatment facility where they were currently living. Participants were interviewed with the Europ‐ASI, and the PDI‐R2, completed the neuropsychological assessments, the PSI full version and HSCL‐10, during the assessment period. We collected data from a large battery of measures, and only selected results are presented in this paper. Estimated time for data collection using the larger test battery was approximately 7 hr per family, and each participant met with the researcher on three to six separate occasions to complete the assessment. Each session lasted between 1 and 2 hr. Data collection for the particular part of the test battery in this study lasted for approximately 4 hr per respondent.

### 2.4 Ethics

The study was approved by The Norwegian Regional Committee for Medical Research Ethics in Eastern Norway (REK‐Øst) and was conducted in accordance with the Declaration of Helsinki of the World Medical Assembly (2004).

### 2.5 Statistical analyses

All analyses were conducted with IBM Statistical Package for Social Sciences (SPSS; versions 22/23/24), IBM Corp. Released 2016. IBM Statistics for Windows, version 24.0. Armonk, NY: IBM Corp. All cases (*N* = 43) were included in the analyses, and there were no missing data. To assess internal consistency, Cronbach *α* coefficients were calculated for all the measurements used in this study. Stress (parental stress, general life stress, and psychological stress), EF (working memory, inhibition, and cognitive flexibility), PRF, and demographics were summarized using descriptive statistics (see Table 1). Bivariate correlations (Pearson's *r*, two tailed) were carried out to study the relationship between the stress variables (parental stress, general life stress, and psychological distress), EF components (working memory, inhibition, and cognitive flexibility), PRF, and demographic variables (age, education, and marital status; see Table 2). To further examine the links between EF and stress components, we carried out two hierarchical ordinary least square regression analyses, using stress components that were significantly correlated with EF (i.e., parental stress and psychological distress but not general life stress) as dependent variables and the EF components (working memory, inhibition, and cognitive flexibility) as independent variables. As education (but not age or marital status) was significantly associated with EF in the correlational analysis, we used education as a control variable. The control variable and the independent variables were entered in two blocks: the first block consisted of education and the second block consisted of the EF components. The analytic strategy allowed us to examine how much additional variance in stress the EF components accounted for before and after controlling for education (see Table 3).

**Table 1:** Descriptive statistics of demographics, substance preference, stress presented by average raw score and standard deviation performance on cognitive tests presented by *T* scores and parental reflective functioning presented by average score and standard deviation

Demographics	*M*	*SD*	Range	*N*	%
Mother's age^a^	31.1	6.4	19–44		
Child's age (months)^a^	8.6	3.8	4–18		
Marital status^a^					
Cohabitant				14	32.6
Partner not cohabitant				7	16.3
Single				22	51.2
Educated (highest completed)^a^					
Not completed primary school				2	4.7
Primary school				24	53.5
High school				12	27.9
University degree				6	4.7
Prefered substance^a^					
Central stimulant				16	37.2
Opioids				14	32.6
Alcohol				7	16.3
Cannabis				6	14.0
Polysubstance abuse^a^				37	86.0
Injecting substances^a^				22	51.2
Overdoses in life^a^					
0				14	32.6
1–5				21	48.9
>5				8	18.5
Prescribed medications^a^					
Medically assisted rehabilitation				11	25.6
Medication for ADHD				4	9.3
Other				8	18.6
Stress					
Parental stress^b^	70.9	19.2	20–98		
General life stress^b^	82.8	18.0	25–99		
Psychological distress^c^	2.5	0.6	1.3–3.5		
Performance on cognitive tests					
Working memory^d^	41.7	8.8	25–65		
Inhibition^e^	39.8	11.3	20–65		
Cognitive flexibility^e^	35.2	11.4	20–63		
IQ^f^					
Total IQ	94.1	14.6	71–125		
Parental RF^g^					
General RF	2.9	1.7	0–6		
Mental health^h^					
HSCL‐10	2.5	0.3	1.3–3.5		
Lifetime PTSD^h^				29	67.4

Abbreviations: ADHD, attention deficit hyperactivity disorder; PTSD, post‐traumatic stress disorder; RF, reflective functioning.

a European Addiction Severity Index, 5^th^ edition.

b Parenting Stress Index, full scale.

c Hopkins Symptom Checklist.

d Letter‐Number Sequencing subtest in the Wechsler Adult Intelligence Scale, 4^th^ Edition.

e Colour‐Word Interference Test, Conditions 3 and 4 from Delis–Kaplan Executive Function System.

f Wechsler Abbreviated Scale of Intelligence Parental Development Interview‐Revised, Reflective Functioning Scale.

g Hopkins Symptom Checklist–10 (HSCL‐10).

h Mini‐International Neuropsychiatric Interview 5.0.0 manual.

**Table 2:** Correlation coefficients between stress (Items 1–3), EF (Items 4–6), parental RF (Item 7), and demographic variables (Items 8–10)

Variable	1	2	3	4	5	6	7	8	9
1.Parental stress^a^									
2. General life stress^a^	.42**								
3. Psychological distress^b^	.57**	.35*							
4. Working memory^c^	−.34*	−.10	−.61**						
5. Inhibition^d^	−.37*	−.10	−.48**	.72**					
6. Cognitive flexibility^d^	−.48**	−.10	−.57**	.64**	.64**				
7. Parental RF^e^	−.49**	−.22	−.56**	.74**	.42**	.58**			
8. Maternal age^f^	−.04	.00	−.01	.12	−.16	.13	−.09		
9. Education^f^	−.19	.15	−.45**	.48**	.34*	.48**	.30	.30	
10. Marital status^f^	−.01	−.07	.20	−.10	.13	−.02	−.09	−.12	−.21

*Note*. *N* = 43.

Abbreviations: EF, executive functioning; NS, no significant differences; RF, reflective functioning.

a Parenting Stress Index, full scale.

b Hopkins Symptom Checklist.

c Letter‐Number Sequencing subtest in the Wechsler Adult Intelligence Scale, Fourth Edition.

d Colour‐Word Interference Test, Condition 3 and 4 from Delis–Kaplan Executive Function System.

e Parental Development Interview‐Revised, Reflective Functioning Scale.

f European Addiction Severity Index.

* *p* < .05.

** *p* < .01.

**Table 3:** Multiple regression analyses for executive functioning (working memory, cognitive inhibition, and cognitive flexibility) predicting stress (parental stress and psychological distress), controlling for education

Variable	*B*	*SE B*	*β*	*R* ^2^	*R* ^2adj.^	*R* ^2change^	*T*	Sig.
Parental stress				.24	.16	.21		.03
Education	0.75	3.69	.03				0.20	.72
Working memory	1.58	0.70	.73				2.27	.98
Inhibition	−0.93	0.45	−.55				−2.08	.60
Cognitive flexibility	−0.25	0.35	−.15				−0.72	.04
Psychological distress				.44	.38	.24		.003
Education	−0.11	0.10	−.14				−1.01	.32
Working memory	−0.03	0.01	−.38				−2.00	.05
Inhibition	0.001	0.01	−.02				−0.12	.91
Cognitive flexibility	−0.02	0.01	−.27				−1.56	.13

*Note*. *N* = 43.

Abbreviation: NS, no significant results.

* **

**Figure 1:**
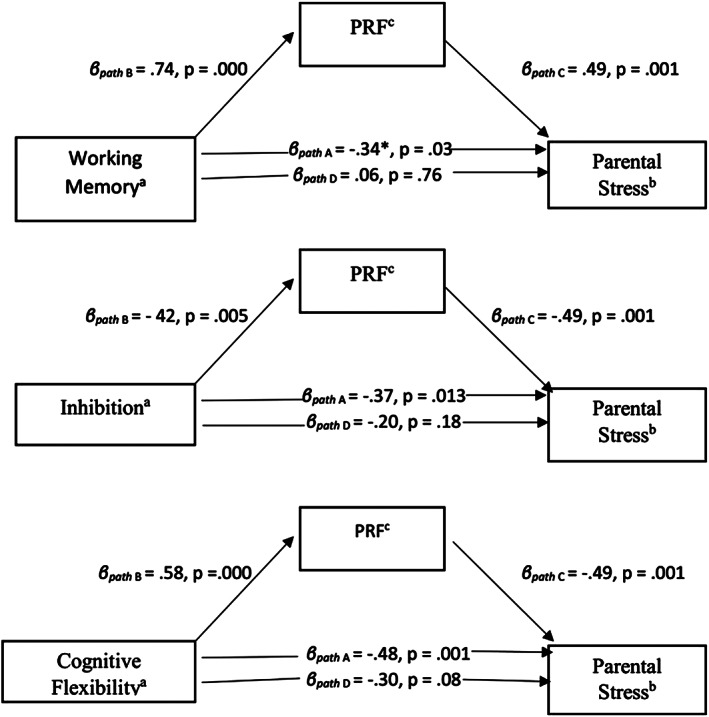
The mediating effect of PRF on the relationship between (a) working memory and parental stress, (b) inhibition and parental stress, and (c) cognitive flexibility and parental stress (*N* = 43). Baron and Kenny's path diagram includes standardized path coefficients that were obtained through a series of multiple regressions to construct the mediation models: Step 1: regression of the dependent variable (parental stress) on the independent variable (executive functioning component: working memory, inhibition, or cognitive flexibility; Path A); Step 2: regression. PRF, parental reflective functioning

**Figure 2:**
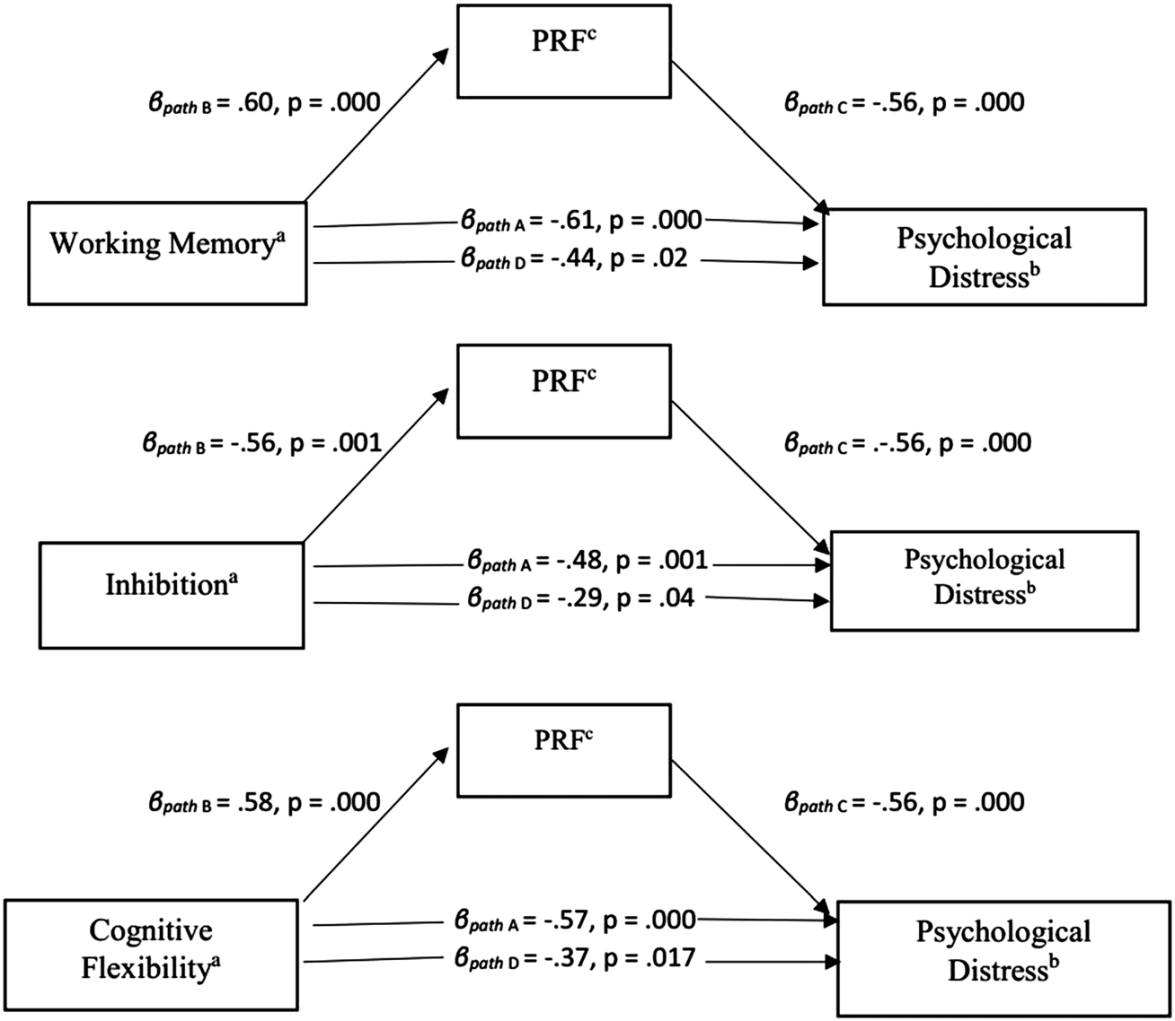
The mediating effect of PRF on the relationship between (a) working memory and psychological distress, (b) inhibition and psychological distress, and (c) cognitive flexibility and psychological distress (*N* = 43). Baron and Kenny's path diagram includes standardized path coefficients that were obtained through a series of multiple regressions to construct the mediation models: Step 1: regression of the dependent variable (psychological distress) on the independent variable (executive functioning component: working memory, inhibition, or cognitive flexibility)

In the second part of the study, mediation models were tested to verify the hypothesis that PRF mediated the relationship between EF (working memory, inhibition, and cognitive flexibility) and stress (parental stress and psychological distress). Due to lack of significant correlations, we did not conduct mediation analyses between the EF components and general life stress. According to the Baron and Kenny (1986) approach, mediation is estimated by using multiple regressions with independent, mediating, and dependent variables. In the present study, EF (working memory, inhibition, or cognitive flexibility) was used as independent variables, PRF was used as the potential mediating variable, and stress (parental stress and psychological distress) was used as the dependent variable. Initially, we regressed the independent variable onto the proposed mediator. Next, we tested the contribution of the independent variable (working memory, inhibition, or cognitive flexibility) across the dependent variable (parental stress or psychological distress). Finally, to investigate mediation, we examined the effect of the EF as an independent variable (working memory, inhibition, or cognitive flexibility) on stress as the dependent variable (parental stress or psychological distress) controlling for the proposed mediator of PRF. Indirect effect tests addressed whether the total effect of the independent variable on the dependent variable were significantly reduced with the addition of the proposed mediator to the model (Preacher & Hayes, 2004; see Figures 1 and 2). The Sobel (1982) test was applied to calculate the indirect effect and its significance using the Indirect.sps tool, version 2.0 Beta, added to the IBM SPSS 25 (see also Preacher & Hayes, 2008).

## 3 RESULTS

Means and standard deviations for all measured variables are presented in Table 1. Measures of working memory, inhibition, and cognitive flexibility show that EF skills were around 1 to 2 *SD* below the average norm (Delis et al., 2001; Weschler, 1999). The entire sample reported experiencing high levels of stress; specifically, general life stress and psychological distress were on average within a clinical range (Abidin, 1995; Strand et al., 2003). Twenty‐one mothers (48.8%) experienced a clinical level of parental stress, and 30 (69.8%) reported experiences of general life stress above the clinical cut‐off level. Thirty‐five mothers (81.4%) experienced psychological distress above clinical cut‐off. The average level of PRF in the whole sample was 2.91 (*SD* = 1.17), which indicates that reflective capacity was low but not completely absent in the group as a whole (Slade, 2005).

### 3.1 Part 1

In the first part of the study, we aimed to investigate associations between EF (working memory, inhibition, and cognitive flexibility) and stress (parental stress, general life stress, and psychological distress). Results are presented in Table 2.

Findings shown in Table 2 indicate that working memory, inhibition, and cognitive flexibility were negatively associated with parental stress. None of the EF components were correlated with general life stress, whereas working memory, inhibition, and cognitive flexibility were negatively associated with psychological distress. PRF was significantly negatively associated with parental stress and psychological distress but not with general life stress. Among the demographic variables, only education was significantly associated with the EF components (working memory, inhibition, and cognitive flexibility) and with psychological distress but not with parental stress or general life stress.

Table 3 presents the results of the multiple regression analysis on parental stress and psychological distress.

Table 3 demonstrates that education only explained 3.5% of the variance in parental stress. Introducing EF (working memory, inhibition, and cognitive flexibility) increased the explained variance to 24.2%, an increase of 20.7% that was significant (*p* = .03). The regression model was significant, *F*(4, 38) = 3.03, *p* = .03, and cognitive flexibility made unique significant contribution (*p* = .04) to variance in parental stress.

Education explained 20.2% of the variance in psychological distress. Introducing working memory, inhibition, and cognitive flexibility in the regression model increased explained variance to 44.2%, an increase of 24% that was significant (*p* = .003). Working memory made a significant unique contribution to the variance in psychological distress (*p* = .05) in the final model that was significant, *F*(4, 38) = 7.51, *p* < .001.

### 3.2 Part 2

In the second part of the study, our aim was to examine whether PRF mediated the relationship between EF (working memory, inhibition, and cognitive flexibility) and stress (parental stress and psychological distress). The inclusion of PRF as a mediator led to a considerable reduction in the effect of the EF on parental stress, working memory (*β* = .06, *t* = 0.30, *p* = .76), inhibition (*β* = −.20, *t* = −1.36, *p* = .18), and cognitive flexibility (*β* = −.30, *t* = −1.83, *p* = .08). The results indicated that PRF mediated the link between each of the EF components individually and parental stress. Standardized coefficients (*β*) for the linear regression analyses are shown in Figure 1. The Sobel test confirmed the significance of causal chains in this complete mediation model between parental stress and working memory (*Z* = −2.51, *p* = .01) and between parental stress and inhibition (*Z* = −2.07, *p* = .04). Although the Sobel test did not significantly confirm full mediation between cognitive flexibility and parental stress (*z* = −1.85, *p* = .06), results were almost significant and indicated a clear tendency towards a mediating effect of PRF (Cohen, 1994; Greenland et al., 2016). In summary, the results of the Sobel test supported the mediation analyses that PRF mediated the relationship between EF and parental stress.

The analyses showed that PRF partially mediated the relationship between working memory and psychological distress (*β* = −.44, *t* = −2.38, *p* = .02), inhibition and psychological distress (*β* = −.29, *t* = −2.13, *p* = .04), and between cognitive flexibility and psychological distress (*β* = −.37, *t* = −2.50, *p* = .02). Standardized coefficients (*β*) for the linear regression analyses are shown in Figure 1. The Sobel test confirmed the significance for partial mediation using PRF as a mediation variable for inhibition and psychological distress (*z* = −2.21, *p* = .03) and cognitive flexibility and psychological distress (*z* = −2.07, *p* = .04) but not for working memory and psychological distress (*z* = −1.27, *p* = .20). The results indicate that PRF significantly affects how cognitive flexibility and inhibition associate with psychological distress.

## 4 DISCUSSION

The first aim of the present study was to examine the relationship between EF (working memory, inhibition, and cognitive flexibility) and stress (parental stress, general life stress, and psychological distress). Poorer EF capacities were associated with experience of higher parental stress, even after controlling for education. A well‐functioning EF system is critical for sensitive caregiving, where mothers have to adapt behaviour to meet environmental demands across multiple contexts and exhibit flexibility in caring for a child (Gonzalez, 2015). Partly supporting our expectations, cognitive flexibility but not working memory showed unique contribution to variance in parental stress. Supporting our results, individual differences in cognitive flexibility have recently been suggested to be important for regulatory capacities associated with perspective taking (Long, Horton, Rohde, & Sorace, 2018). Previous studies have found that cognitive flexibility is impaired in individuals with SUD (Cunha et al., 2010). In addition, impairments in cognitive flexibility are associated with a heightened experience of stress in parents (Sturge‐Apple, Jones, & Suor, 2017) and with emotion dysregulation in women with SUD (Marceau et al., 2018). Mothers with young children and mothers with SUD are shown to have a readily activated “hot” EF system (i.e., EF “coloured” by emotion) when faced with distress (Gladwin & Figner, 2014; Gonzalez, 2015; Volkow & Baler, 2014). It is possible that weaker capacities in cognitive flexibility made it particularly difficult for the mothers in our study to tolerate the demands of a dyadic focus, switching between self and the child and between activities outside and inside the dyad, hence experiencing a heightened parental stress level. Indeed, mothers with SUD, interacting with their children, are shown to be more prone for stress compared with mothers without SUD (Rutherford, Williams, Moy, Mayes, & Johns, 2011). For example, a child demanding attention or an infant crying seems to trigger stress reactivity rather than reward salience in mothers with SUD (Rutherford et al., 2011). In addition, mothers with SUD exhibit reduced activation in reward regions of the brain when they are observing their own children (Kim et al., 2017). Neurobiological evidence shows that reduced prefrontal functioning, related to EF capacities, is associated with increasing levels of stress (Li & Sinha, 2008). Together, these studies might help explain the effect of EF and particularly cognitive flexibility on parental stress seen in the mothers in our study. We suggest that our group of mothers with weak cognitive flexibility capacities were less able to access adequate regulation strategies associated with a heightened experience of parental stress.

As expected, poor EF capacities were also significantly associated with heightened psychological distress in mothers in this study. Supporting our results, differential vulnerability to internal demands has been found to affect distress tolerance (Belsky, Bakermans‐Kranenburg, & Van IJzendoorn, 2007). Reduced distress tolerance is considered a central component of SUD (Li & Sinha, 2008; Tronick et al., 2005). Previous studies have demonstrated that mothers with SUD have a heightened risk for difficulties in emotion regulation capacities (Suchman et al., 2012), which in turn relates to reduction in distress tolerance (Daughters, Lejuez, Kahler, Strong, & Brown, 2005; Deater‐Deckard et al., 2016; Leyro, Zvolensky, & Bernstein, 2010). Among the EF components, working memory showed a unique contribution to variance in psychological distress in the mothers after controlling for education. Our results indicate that the ability to keep information in mind and integrate current content with new information might be particularly important for managing psychological distress in mothers with SUD. Supporting our results, weak working memory skills has been associated with difficulties in emotion regulation strategies in mothers (Rutherford, Booth, Crowley, & Mayes, 2016; Sheinkopf et al., 2005). Therefore, it is possible that high psychological distress adversely affects parental stress.

Surprisingly, general life stress was not significantly associated with any of the EF components. Contrary to the results in our study, previous research has found that EF impairments are associated with experience and management of general life stress (Hofmann et al., 2012; Koenig, Walker, Romeo, & Lupien, 2011; Schmeichel & Tang, 2014; Williams et al., 2009). Further, EF has been found to be associated with PRF in mothers with SUD (Håkansson et al., 2017; Rutherford et al., 2017; Yatziv et al., 2018), and we suggest that EF capacities and deficits might affect relational forms of stress (parental stress and psychological distress) and particularly in mothers with SUD because of a possible mediating functioning of PRF. Indeed, these associations between EF and general life stress may not be present as general life stress is less relationally focused compared with parental stress and psychological distress.

Our second aim in the study was to enhance our understanding of the role between EF (working memory, inhibition, and cognitive flexibility) and stress (parental stress and psychological distress) with PRF as a mediator. Congruent with our hypothesis, the results confirmed a clear mediating effect of PRF in the association between EF (working memory, inhibition, and cognitive flexibility) and parental stress. In other words, EF might have indirectly influenced parental stress via the capacity to mentalize (i.e., PRF). Poor PRF might lead mothers to be more vulnerable to parental stress because of weaker EF capacities. In contrast, adequate PRF capacities might have strengthened mothers' regulation capacities, leading to access of EF in a more helpful way when facing demanding parental situations. Supporting our results, findings from previous studies have suggested PRF as a core capacity in regulating strong emotions when confronted with relational stress, including parental stress (Fonagy& Bateman, 2016; Slade, 2005). Furthermore, mothers with weaker PRF capacities that demonstrate difficulties reflecting around their child's mind and with a low capacity to tolerate demands from the child have been found to exhibit decreased tolerance of stress (McQuillan & Bates, 2017; Rutherford et al., 2015). Together, previous research and our results indicate that the mediating effect of PRF in the association between EF and parental stress might affect the parent–infant relationship.

We also found that PRF mediated the relationship between EF (working memory, inhibition, and cognitive flexibility) and psychological distress but to a lesser degree than parental stress. It likely that other variables in combination with PRF constituted mediators between EF and psychological distress. Impairments in perspective taking, a capacity fundamental for PRF, have previously been associated with high psychological distress (Allen & Fonagy, 2002). In addition, a recent study found that low PRF heightens stress sensitivity in mothers with mental health problems (Krink et al., 2018). Numerous studies have demonstrated that mental health issues adversely affect RF (Borelli, West, Decoste, & Suchman, 2012; Conklin, Bradley, & Westen, 2006). Our results indicate that EF associated with psychological distress partly via poor or adequate PRF. Interestingly, the Sobel (1982) test showed that PRF partially mediated the relationship between inhibition, cognitive flexibility, and psychological distress but not between working memory and psychological distress. As the regression showed that working memory was particularly related to psychological distress, but not mediated by PRF, it is likely that working memory is more directly associated with psychological distress.

Recent studies have suggested that individual capacities in allocating EF in the face of stress work in a dynamic manner, where individuals with deficits in EF might have a limited capacity for stress tolerance (Kluwe‐Schiavon et al., 2016). We suggest that the experience of parental stress and psychological distress in the mothers in our study could be heightened because of a pre‐existing weak EF system in combination with deficits in PRF. Indeed, mothers with adequate PRF could have had enhanced capacity to access EF during demanding intrarelational and interrelational situations (e.g., parental stress and psychological distress) because they were able to reflect upon them (e.g., having adequate PRF) and therefore were more able to regulate stress in demanding contexts concerning internal or relational situations.

### 4.1 Limitations and strengths

First, based on our theoretical focus, we have tested one model regarding associations between EF, PRF, and stress. However, no single model can fully predict reality, and our model is one out of many possible approaches. In addition, the reliance on a cross‐sectional design precludes inferences about causality. Future research with prospective or longitudinal designs could determine the direction and temporal order of relationships among the variables. Second, self‐report data could generate participant bias, and future studies should include physical measurements of stress. Third, results of the current study may have been influenced by unmeasured confounding variables. For instance, we did not include details about SUD, such as preference for a particular substance or severity of dependence (Pajulo et al., 2012). We did not include specifics about mental health diagnosis, including post‐traumatic disorder, or developmental trauma, both of which have been found to affect PRF, EF, and stress (Augusti & Melinder, 2013; Briere, Kaltman, & Green, 2008; Cromer & Sachs‐Ericsson, 2006; Håkansson, Watten, Söderström, Skårderud, & Øie, 2018). Fourth, our sample size was rather small, although within the norms for this kind of study (Pajulo et al., 2012; Suchman, Decoste, Mcmahon, Rounsaville, & Mayes, 2011). Because the Sobel (1982) test relies on the assumption of normal‐distributed samples, a small sample size may have underestimated the mediation effect. To increase statistical power, our study should be replicated using a larger sample size.

To our knowledge, this is the first study to investigate associations between EF and different types of stress in mothers with SUD to small children. In addition, we do not know of any previous studies that have investigated PRF as a mediator between EF and stress. Our research therefore extends on previous theoretical and clinical knowledge in the field. Furthermore, we conducted semistructured interviews and administered a selection of measures with strong psychometric properties. In addition, all the mothers completed the full assessment battery. Mothers with SUD are often considered particularly vulnerable in the parental role, in addition to being difficult to offer appropriate, customized interventions (Pajulo, Suchman, Kalland, & Mayes, 2006). They are also vulnerable to intergenerational transmission of risk (Håkansson et al., 2018; Kelly, Slade, & Grienenberger, 2005) and therefore are an important population to offer targeted effective interventions. The results of our study indicate that there are dynamic processes between EF, PRF, and the experience of stress. Targeting individual capacities and vulnerabilities in these components might help overcome some of the difficulties in developing effective interventions, and therefore, results of our study may be useful when considering the development of psychotherapeutic interventions.

## 5 CONCLUSIONS AND CLINICAL IMPLICATIONS

The findings from this study suggest that parental stress and psychological distress in mothers with SUD should be understood within the context of EF, with PRF as a mediating variable. On the basis of our results, we suggest that it is important to consider individual differences in mothers with SUD, particularly in PRF and EF capacities, before developing interventions. Individually customized interventions that targets reflective capacities, such as mentalization‐based therapies (Sadler et al., 2013; Suchman et al., 2017), dialectal behaviour therapy (Neacsiu, Bohus, & Linehan, 2014), or mindfulness‐based interventions (Short et al., 2017) might lead to improvements in accessing EFs and reduce the experience of parental stress and psychological distress. In addition, interventions directly targeting EF capacities, particularly cognitive flexibility and working memory, could lead to increased emotional regulation capacity in the mother, which could then provide the foundations for her to access subsequent relationally based psychotherapeutic interventions.

## CONFLICT OF INTEREST

The authors declare they have no competing interests.

## FUNDING

The project received financial support from The Research Council of Norway (NFR) Grant 213079/H10 and from Innlandet Hospital Trust.

## Footnotes

*
*p* < .05.

**
*p* < .01.
